# Concussion knowledge, attitudes, and norms: How do they relate?

**DOI:** 10.1371/journal.pone.0282061

**Published:** 2023-02-22

**Authors:** Aliza K. Nedimyer, Avinash Chandran, Melissa K. Kossman, Paula Gildner, Johna K. Register-Mihalik, Zachary Y. Kerr

**Affiliations:** 1 Department of Exercise and Sport Science, University of North Carolina at Chapel Hill, Chapel Hill, NC, United States of America; 2 Matthew Gfeller Center, University of North Carolina at Chapel Hill, Chapel Hill, NC, United States of America; 3 Human Movement Science Curriculum, University of North Carolina at Chapel Hill, Chapel Hill, NC, United States of America; 4 Datalys Center for Sports Injury Research and Prevention, Indianapolis, IN, United States of America; 5 School of Health Professions, University of Southern Mississippi, Hattiesburg, MS, United States of America; 6 Injury Prevention Research Center, University of North Carolina at Chapel Hill, Chapel Hill, NC, United States of America; University of A Coruna: Universidade da Coruna, SPAIN

## Abstract

**Background:**

Relationships between the constructs of concussion-related knowledge, attitudes, and norms and their influence on observed care-seeking behaviors have previously been examined. Current models posit that these constructs serve as potential mediators of care-seeking behaviors; however, the dynamics between them have yet to be reconciled.

**Methods:**

A cross-sectional, online survey explored relationships among the latent constructs of concussion-related knowledge, attitudes, and norms in parents of middle school children who participate in sports in multiple settings. A just-identified and two overidentified path models were explored and compared in an effort to understand such relationships.

**Results:**

A total of 426 parents of United States middle school students were surveyed and included in analyses (mean age = 38.7±9.9 years; 55.6% female; 51.4% white/non-Hispanic; 56.1% with at least a bachelor’s degree). All parents had middle school aged children who participated in sport in both the club and school settings. The best fitting model was a just-identified model with concussion-related norms influencing concussion-related knowledge and attitudes, and concussion-related knowledge influencing attitudes. This model accounted for 14% of the variance in attitude and 12% of the variance in knowledge.

**Conclusions:**

Study findings suggest that the constructs of concussion-related knowledge, attitudes and norms are directly related to one another, yet the dynamics of such relationships may be complex. As such, a parsimonious interpretation of these constructs may not be appropriate. Future research should work to further reconcile the dynamics between these constructs, and the impact these dynamics may have in influencing care-seeking behaviors beyond serving as mediators.

## Introduction

It is estimated that approximately 1.1–1.9 million sport- and recreation-related concussions occur among children under 18 years of age in the United States annually [1]. Much of the current research related to concussion examines high school and collegiate athletes (typically aged 15–22 years), or is conducted within emergency departments and recreational sport leagues [2–4]. While some research in other settings, such as schools and primary care clinics has been conducted, it remains limited therefore limiting knowledge related to those seeking care in these settings. As such, Middle school (MS) aged athletes remain an understudied population related to concussion and concussion reporting. MS sports and MS athletes refers to a subset of the general population comprised of students aged 10–15 years. While the specific grade ranges of MS vary by location, it generally includes students in grades 6 through 9. Due to the age of MS student athletes, parents play an important role in decision making related to their medical care. While this is true with all minors, it is particularly salient in the MS aged population as they require transportation to medical visits typically provided by parents, along with the fact that MS aged children may be less medically literate than older children. Therefore, it is important to include these parents and consider their perspectives when researching this subset of the population.

Previous concussion-related research has worked to understand various aspects of the injury, including descriptive epidemiology in various settings [2–4], acute care and assessment [5, 6], rehabilitation and recovery patterns [7, 8], and long-term effects [9, 10]. Such existing research has also aided the development and deployment of concussion prevention and education interventions [11, 12]. Additionally, previous research has explored concussion care-seeking intentions and reporting behaviors, as well as ways to promote these actions [13–15]. Much of the research related to concussion care-seeking intentions and reporting behaviors is framed using social constructs. These commonly used constructs include knowledge of concussive injuries, attitude toward concussive injury and care seeking behaviors, and social norms surrounding both concussive injury and care seeking behaviors. This social dimension of concussive injury, including the constructs listed here, have been previously measured in numerous ways [10, 13, 15]. The Theory of Planned Behavior (TPB) is one framework that has been employed to explore some of these constructs [16, 17]. The TPB posits that beliefs ultimately drive behavior, and that the best predictor of a given behavior is the intention to participate in or complete a given behavior [16–18]. The TPB additionally theorizes that intentions are influenced by perceived behavioral control, attitudes, and subjective norms regarding the behavior in question [16–18]. Further, the stronger the intention, the more likely the individual is to complete or participate in such behavior [16–18].

Related to concussion, the TPB has been used understand how concussion-related knowledge, attitudes, and norms may influence concussion reporting [13–15]. Beyond knowledge and attitudes, norms indicate cultural expectations or standards of social behavior. Related to concussion, these include perceived stakeholder support for concussion reporting related to reporting and encouraging reporting, as well as missing practices and games due to concussive injury, along with other expectations and behaviors. Recent research pertaining to concussion suggests attitudes toward concussion reporting are associated with reporting intentions and prior care-seeking behaviors surrounding concussive injuries [19], as well as parental intentions surrounding their child’s participation in contact sport [20]. However, previous studies also suggest higher levels of concussion symptom knowledge may not always be indicative of safer attitudes toward concussion [21]. As such, it is possible that simpler relationships between these variables that have yet to be reconciled may exist, and therefore impact concussion reporting.

Although previous work suggests concussion-related knowledge, attitudes, and norms serve as potential mediators of behaviors related to concussion reporting [14, 22–24], the nuanced relationships between these constructs, and the impacts they have on each other and concussion reporting more broadly, have yet to be fully reconciled. The relationships among these constructs and their role in concussion reporting must be properly and fully understood before these constructs can be used moving forward in the creation and implementation of existing and new concussion education efforts. Further, because of the role parents play in influencing and providing medical care to MS aged athletes, it is important these constructs be explored within this population. Thus, our study aims to explore, and begin to define, the relationships among the latent constructs of concussion-related knowledge, attitudes, and norms using a nationwide survey sample of parents of MS children. Based on previous research findings and the TPB [13, 14, 16–18, 25–27], we hypothesized that concussion-related norms would influence concussion-related knowledge and attitudes in parents of MS athletes. The potential for simpler existing relationships between these variables that have yet to be reconciled serves as the foundation for our work. Once better understood, these relationships will allow for modification and advancement of targeted concussion education programs.

## Methods

### Design

This study utilized a cross-sectional survey design. The survey instrument was distributed online to parents of MS aged athletes throughout the United States. Prior to distribution of the survey, the study was approved by the Institutional Review Board at The University of North Carolina at Chapel Hill.

### Participants and sampling

The study sample was part of a larger sample collected using Survey Sampling International (SSI), a large research firm that helps to connect marketers and researchers with consumer insights. The SSI company recruited a pool of United States residents that had previously agreed to participate in online, survey-based research. SSI maintains certification processes to ensure data quality, and, based on SSI’s process, those eligible for specific studies can be identified. For the present study, the participant pool recruited by SSI included only individuals who self-reported being parents of a child aged 10–15 years. No other exclusion criteria were utilized in an effort to provide a range of parents. SSI then randomly generated a sample from this pool that were subsequently invited to participate in this study. Study specific information was not included in the initial invitation to avoid self-selection bias. Rather, parents agreed to participate in the study and were then given more specific details. Those that agreed to participated did, and following their participation in the study, SSI reimbursed participants with “reward points” that they could then redeem for various incentives. Ultimately this allowed for the collection of a large, random, sample of MS parents from throughout the United States.

### Data collection

The survey instrument utilized was based on a modified version of an existing instrument [19, 28]. During development of the current instrument, input was sought from injury epidemiologists, athletic trainers, sports medicine physicians, and parents of youth sport athletes. Initial pilot testing was completed with a sample of parents of MS children. Following pilot testing and expert input, the survey instrument was revised to reflect their suggestions and this final version was deployed.

Data were collected electronically using Qualtrics software (Qualtrics^XM^, Provo, UT). A panel of 1362 randomly selected US residents aged 18 years and older who identified as parents of children between the ages of 10–15 years were invited to participate and agreed to complete the survey instrument. Prior to completing the survey, parents provided written informed consent. Of these 1362 individuals who consented to participate, 426 indicated their children were currently enrolled in MS, participated in sport in both the school and club settings, and completed all pertinent questionnaire items required for analysis for the current study (Table 1). Parents with children participating in sport in both the school and club settings were utilized to reconcile the dynamics of these latent constructs in those with multidimensional sport exposures.

**Table 1:** Descriptive profile of parents of middle school students (n = 426).

Sample Characteristics	n	%
Sex		
Male	189	44.4
Female	237	55.6
Age		
Under 30	59	13.9
30–39	183	43.0
40–49	120	28.1
50–59	51	12.0
60 and over	13	3.0
Race/Ethnicity		
White/Non-Hispanic	219	51.4
Other^a^	207	48.6
Highest Level of Education Completed^b^		
At least a bachelor’s degree	239	56.1
Less than a bachelor’s degree	187	43.9
Sport Participation of Students^c^		
Non/Limited Contact Sports^d^	47	11.0
Contact Sports^e^	269	63.2
Football	110	25.8

^a^ Respondents were requested to select which race/ethnicity they identified with. Reponses were collapsed into White/Non-Hispanic or other, which included Black/African-American, Hispanic/Latino, Asian/Pacific-Islander, or Mixed race/other.

^b^ Respndents were asked to provide the highest level of education they had completed. At least a bachelor’s degree included the options of Bachelor’s degree, Master’s degree, Doctorate, or Professional degree. Less than a bachelors degree included less than high school, high school or GED, some college—no degree, or Associate’s degree.

^c^Includes organized sports played at middle school or youth club/recreation leagues in the last year. Contact level classifications originate from Rice et al. [32] If children played multiple sports, they were categorized into the highest contact level group (e.g., a child participating in ice hockey and tennis was classified in the “contact sports” category).

^d^Includes archery, baseball, cross country, dance, fencing, flag football, golf, racquetball, softball, swimming, tennis, track and field, and volleyball.

^e^Includes basketball, boxing, cheerleading, field hockey, gymnastics, ice hockey, lacrosse, martial arts, soccer, water polo, and wrestling.

### Measures and statistical analyses

Outcomes of interest were concussion-related knowledge, attitudes, and norms. Concussion symptom knowledge was examined using 25 yes/maybe/no items related to concussion symptoms (Cronbach *α* = 0.90). Correct answers scored two points, ‘maybe’ answers scored one point, and incorrect answers scored zero points. Resulting scores ranged from 0–50, with higher scores indicating better, or more, knowledge. Concussion care-seeking attitudes were measured with five items on a 7-point Likert scale, resulting in a total range of 7 to 35, with higher scores indicating better (i.e., more positive, favorable, safer) attitudes (Cronbach *α* = 0.89). Concussion-related norms were measured using 19 items on a 7-point Likert scale (Cronbach *α* = 0.89). Participants completed 8 items related to MS sports, 8 items related to club sports, and 3 items related to concussion and sport participation in general, for a total of 19 items. Five items (2 related to MS sports, 2 related to club sports, and 1 related to concussion and sport participation) were reverse coded such that higher scores indicated more positive norms (total scores ranged from 7 to 133). Norms assessed surrounded perceived stakeholder support for concussion reporting related to reporting and encouraging reporting, as well as missing practices and games due to concussive injury. For example, more positive norms related to concepts of there being more support for, or a culture that encourages, reporting injuries and taking time off when an injury has been sustained, among other actions.

**Fig 1:**
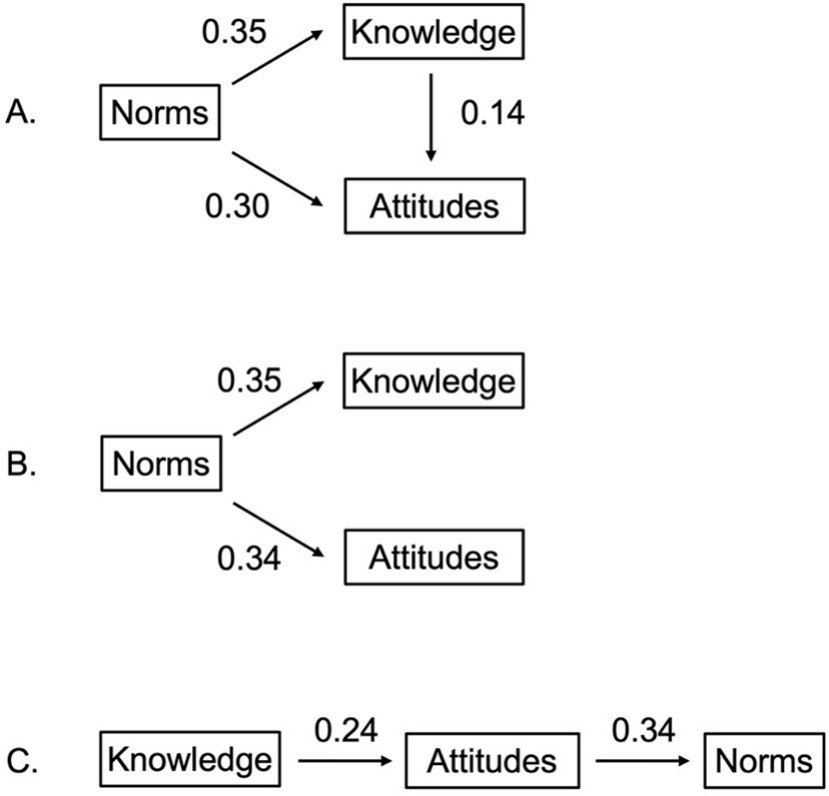
Models examined with standard estimates. A. Just identified model. B and C. Overidentified models.

Descriptive characteristics of categorical variables were examined using frequencies and percentages; continuous variables were examined using means and standard deviations or medians and interquartile ranges (IQRs) for variables that were not normally distributed. Following this, path analysis techniques were used to explore the relationships among concussion-related knowledge, care seeking attitudes, and norms in parents of MS children. The TPB and existing literature was used to inform a theoretical model describing the relationship between concussion-related knowledge, attitudes, and norms [13–15]. We hypothesized a just identified specification of relationships between the aforementioned variables, with knowledge influencing norms and attitudes, and attitudes influencing norms (Fig 1A). Just identified models are considered saturated, meaning there is one way to solve for each parameter, and included direct paths to all variables. Following this, a pair of overidentified alternative models were explored and tested for a more parsimonious expression of the relationships between these factors (Fig 1B and 1C). Overidentified models are not saturated, at least one parameter can be solved for in multiple ways, and ultimately do not include direct paths to all variables. Rather, paths have been removed, single variables influence multiple others, or paths allowing one variable to intervene on another are included [29].

Maximum likelihood estimation was used for estimation of the proposed model parameters, and model fit was evaluated using multiple assessments. These assessments included the following: 1) chi-square statistics, which tested the null hypothesis that any given just-identified model parametrized model covariance appropriately given the observed data (with statistical significance indicating there were sufficient discrepancies between the implied model covariances and the observed sample covariances–in which case the overidentified model was considered to be the most appropriate alternative) [30]; 2) the Bentler Comparative Fit Index (CFI; values range from 0–1, with higher values of indicating better fit); 3) standardized root mean square residual (SRMSR; smaller values indicating a better fit, with 0 being a perfect fit); and 4) root mean square error of approximation (RMSEA; ranging from 0–1, with lower values indicating better model fit) [31]. Data were analyzed using SAS version 9.4 (SAS Institute Inc., Cary, NC).

## Results

### Demographics

**Fig 2:**
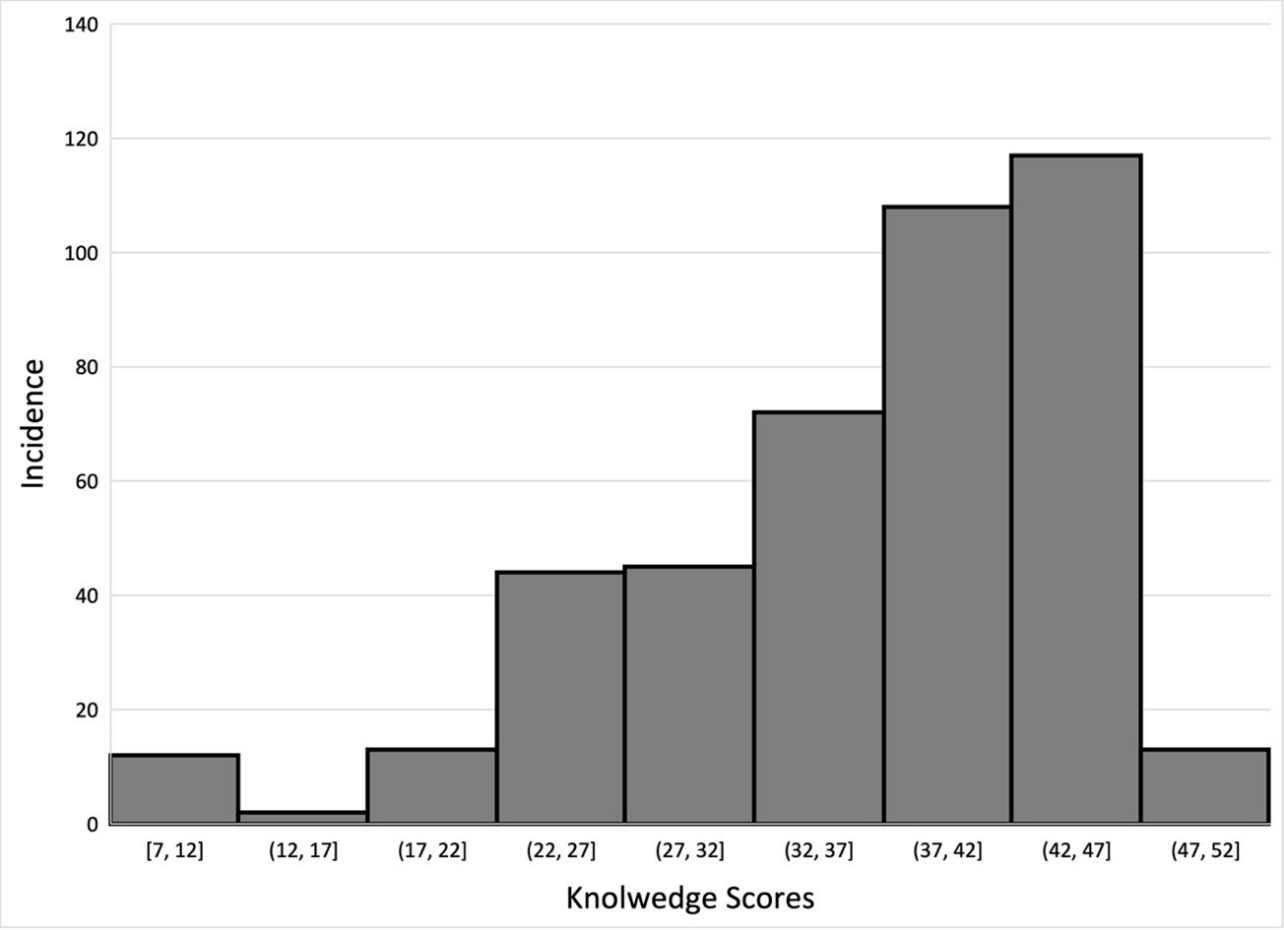
Distribution of knowledge scores.

**Fig 3:**
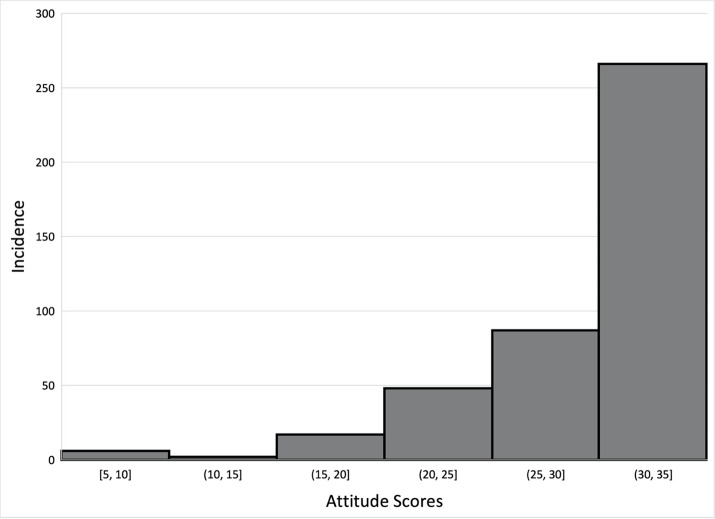
Distribution of attitude scores.

**Fig 4:**
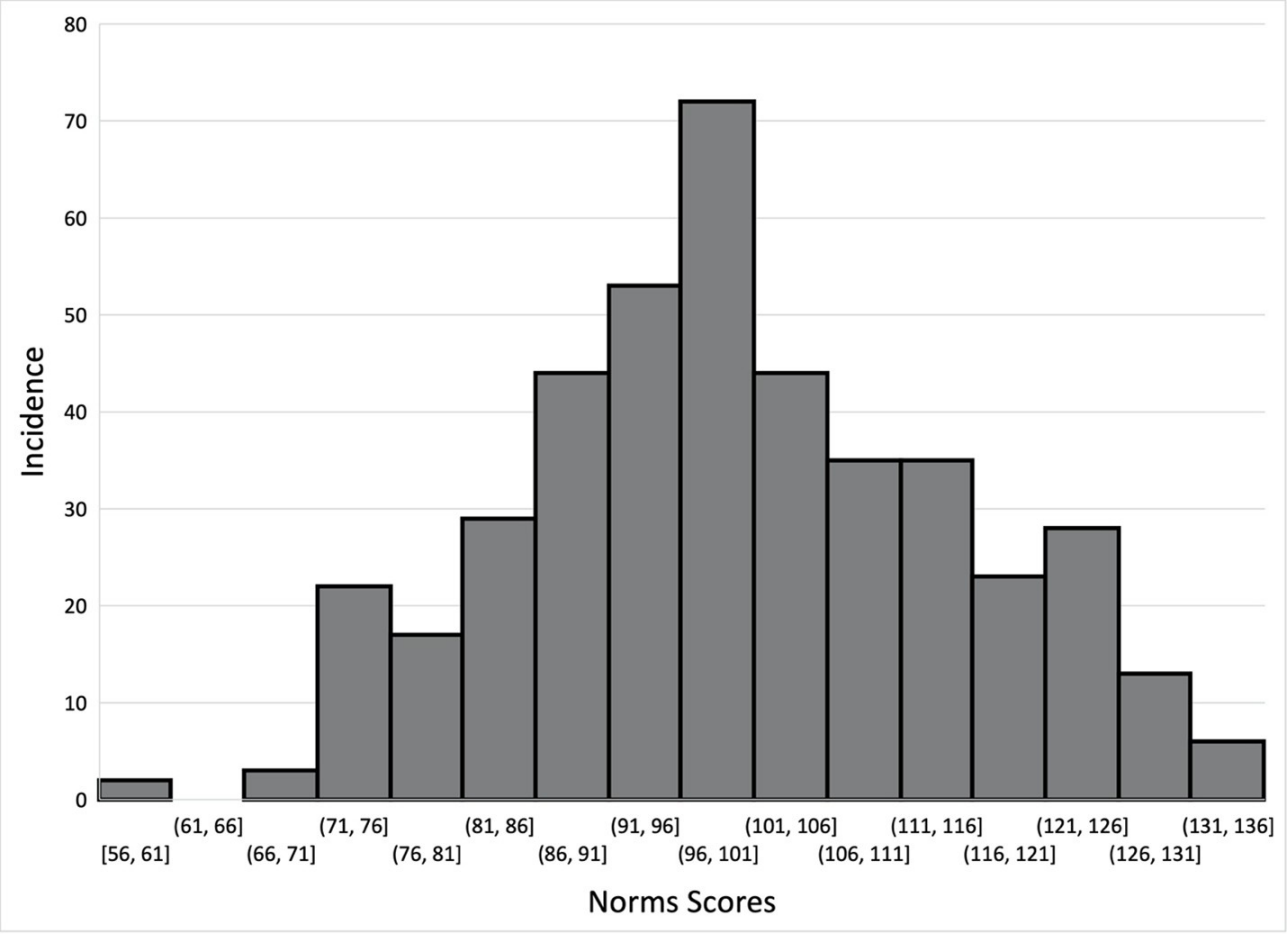
Distribution of norm scores.

Parents of United States MS students (n = 426) were surveyed and responded to all questions related to variables of interest. These parents had a mean age of 38.7 ± 9.9 years and 55.6% were female. Of these parents, 51.4% identified themselves as white/non-Hispanic and 56.1% completed at least a bachelor’s degree (Table 1). The median knowledge score was 39.0/50 with an IQR of 12.0, the median attitude score was 32.0/35 with an IQR of 7.0, and the median norm score was 100.0/119 with an IQR of 21.0 (Figs 2–4). Distributions of concussion-related knowledge and attitude scores were skewed left, (Figs 2 and 3), while concussion-related norms scores were normally distributed (Fig 4). The MS children of surveyed parents participated in a wide variety of sports within both the school and club setting (Table 1).

### Path analysis models

#### Just identified model

Based on the TPB, an initial just-identified model (including direct paths between all constructs) explored concussion-related norms influencing knowledge (standard estimate = 0.35) and attitudes (standard estimate = 0.30), with knowledge also influencing attitudes (standard estimate = 0.14) (Fig 1A). This model explained 14% of the variance in attitude and 12% of the variance in knowledge. As anticipated given the just-identified nature, this model fit the data perfectly and had the following fit statistics: χ^2^ = 0, CFI = 1.0, and SRMSR = 0.0.

#### Overidentified models

Two overidentified models were examined as parsimonious alternatives to the just-identified models described above. These models did not include direct paths to all variables (Fig 1B and 1C). First, we examined a specification in which concussion-related norms directly influenced concussion-related knowledge (standardized estimate = 0.35) and attitudes (standardized estimate = 0.34). This model explained 12% of the variance in knowledge and 11.9% of the variance in attitudes. In further inspecting model fit, we observed this model not to be superior to the just-identified alternative (χ^2^ = 8.74; p = 0.0031). Remaining fit statistics for this model were considered adequate, including the RMSEA estimate (RMSEA = 0.13), the SRMSR (SRMSR = 0.05), and the CFI (CFI = 0.93). We also considered an alternative overidentified model in which knowledge influenced attitudes (standard estimate = 0.24), and attitudes influenced norms (standard estimate = 0.34). This model explains 6.0% of the variance in attitudes and 11.9% of the variance in norms. This model also appeared to be a not as well fitting alternative to the just-identified model (χ^2^ = 36.5; p < 0.001), with a poor RMSEA estimate (RMSEA = 0.29), a high SRMSR (SRMSR = 0.11), and a CFI = 0.69.

## Discussion

To our knowledge, this is the first study to examine a literature and theory-driven understanding of the relationships between the latent constructs of concussion-related attitudes, knowledge, and norms. Based on previous research findings and the TPB [13, 14, 16–18, 27], we hypothesized that concussion-related norms would influence concussion-related knowledge and attitudes in parents of MS athletes. Study results indicate that a parsimonious interpretation of these constructs may not be appropriate, indicating that while these constructs are related, the dynamics of such relationships may be complex. For example, study results found the best fitting model in this population to be the just-identified model in which concussion-related norms influence concussion-related knowledge and attitudes toward care seeking, with concussion-related knowledge also influencing care seeking attitudes. With an understanding that concussion-related knowledge may not be the only construct influencing concussion-related attitudes, there is an indication that creating concussion education materials that intervene specifically on concussion-related norms may be beneficial. Such norms, or social and cultural expectations related to such injuries may also impact concussion-related knowledge and care seeking attitudes. These specific relationships observed in the models have the potential to impact the way that concussion education strategies are structured and delivered to both parents of MS athletes, as well as others, ultimately improving concussion recognition and care.

Descriptive analysis of our data generally suggested that parents of MS athletes who participate in sport in both the school and club settings have a high knowledge of concussion symptoms, as well as mostly positive attitudes toward concussion care-seeking (Figs 2 and 3). These data are skewed to the left, indicating higher concussion-related knowledge and more positive concussion-related attitudes as they pertain to care seeking, which is consistent with the existing literature [21, 33, 34]. While this study did not explore where parents obtain this knowledge, or what is influencing the development of such care seeking attitudes, existing research indicates that parents obtain such information from healthcare providers as well as the media and internet [35–38]. Additionally, parents whose children participate in MS sports in solely the school or the club setting may have differing levels of concussion-related knowledge or different concussion-related attitudes toward care seeking than those whose children participate in sport in both settings [39]. While existing research indicates disparities in coaching education related to concussion exist between club and school sports at the high school level [40], it is plausible that there are likely differences between the education of both parents and athletes in the settings, along with a lack of information being transferred by coaches who are receiving less or no education on concussion. Further research should investigate whether MS or club settings are providing impactful concussion education to their athletes and their athletes’ parents along with coaches.

Concussion-related norm scores were more normally distributed than the knowledge and care seeking attitude scores, indicating more variation in perceived concussion-related norms among the parents included in this study (Fig 4). This variation may be due to the measurement scale used or the norms that were assessed, including those related to culture surrounding perceived stakeholder support for concussion reporting related to reporting and encouraging reporting, as well as missing practices and games due to concussive injury. However, it may also be a result of variation in the MS sport setting, as various stakeholders in the school and club settings likely behave differently leading to the development of different sport cultures and social expectations, ultimately norms. As such, it is important to understand what drives the development of these norms surrounding concussion reporting and care in various settings, and how they may impact concussion-related knowledge and care seeking attitudes. With the just identified and one of the overidentified models indicating that concussion-related norms have a direct, positive influence on concussion-related knowledge and attitudes, it is evident that cultural and social expectations that create concussion norms should not be ignored. As such, targeting the development of concussion-related norms within this population and setting may be helpful in improving concussion-related knowledge and attitudes. However, these models also indicate that such relationships are dynamic in nature and should be further explored in this population of parents of MS athletes, as well as in other populations based on sport participation or level of play, as variation likely exists.

To date, many educational interventions have targeted concussion-related knowledge and attitudes, directly targeting these two constructs individually [13, 14, 22, 23]. Strategies utilized to create and deploy such interventions, both previously and currently, have been wide ranging in nature. They commonly involve some sort of educational presentation made by the athletic trainer or athletic director at preseason meetings. However, it is important to note that access to on-site healthcare and injury prevention specialists such as an athletic trainer is uncommon at the middle school level. Other educational strategies that have been employed include videos [41], interactive training programs for coaches [42, 43], and utilization of parents to educate one another [12]. However, previous studies have found that increasing parents’ concussion knowledge may not improve their ability to identify potential concussions in their children when they occur [44], and that increased levels of concussion knowledge may not be indicative of more positive attitudes toward concussion [21]. Although limited in the current literature, there may exist additional benefit from targeting or including concussion-related norms in concussion education and prevention strategies to improve safety, as study findings indicate that there exists a relationship between concussion-related norms, knowledge and attitudes in which norms may influence the other constructs. Additionally, there exists some indication that due to the dynamic nature of these relationships, individually addressing these constructs of concussion-related knowledge and attitudes may not be the most effective manner for improving them, and therefore improving concussion safety in general.

A focus on concussion-related knowledge, attitudes, and norms in concussion education and prevention strategies has the potential to impact concussion safety on multiple levels, including efficiency. While some studies have started to look at external influences on concussion-related knowledge and attitudes, such as interpersonal relationships including teammates, coaches, and parents [15, 24, 45], there still remain other factors that have the potential to influence concussion-related norms, which may also influence concussion-related knowledge and attitudes. While concussion-related knowledge and attitudes are mostly directed at the intrapersonal level, norms have the potential to impact the interpersonal, community, and organizational levels. Although the utilization of the TPB and other existing research as a guide has been helpful for impacting concussion-related knowledge and attitudes at the intrapersonal level, given that concussion-related norms may influence concussion-related knowledge and attitudes, leveraging the socioecological framework to help target concussion education and prevention may prove to be more effective. This framework has previously been posited as a guide for a multifactorial approach to concussion education and prevention [44], as it takes into account numerous levels of influence on behavior, many of which are interpersonal or context-based (i.e. organization or community level) [44, 46]. As such, education strategies that work to create culture change at organizational and community levels will also work to change the concussion-related norms that are upheld by that specific organization or community. Due to the dynamic nature of the relationship between these constructs, such impacts may also influence the concussion-related knowledge and attitudes of individuals within a community.

It has additionally become apparent that while intervening on each of these constructs in isolation may be beneficial, there may also exist effective ways to improve concussion safety that encompass interventions and educational programs targeted at individual constructs in a structured way, as well as targeting multiple constructs simultaneously. As such, there exists a need to understand the complex dynamics in an effort to approach concussion education in a multifactorial manner. This multifactorial approach may also need to include multiple levels of the socioecological framework, as some constructs lend themselves more closely to one level over another. Using a multifaceted, multilevel approach allows for both culture change and individual change as it relates to the concussion-related knowledge, attitudes, and norms in parents of MS athletes.

### Limitations

It is important to understand the inferential limitations of the present study, the largest of which being its generalizability. While the utilization of a nationwide sampling pool attempted to collect a representative sample, it is important to note that our findings may not be generalizable to all MS parents, nor the parents of youth or high school sport athletes. Additionally, as with all survey-based research there exists the risk of social desirability and recall bias. Our models are also limited by sample availability and the characteristics of the variables examined. Further, the models included here were hypothesized and tested on the basis of existing literature and theoretical support. It is probable that alternative models may result in better fit, but such models were not tested in an effort to remain consistent with the theoretical underpinnings of this study. Future research should work to further reconcile the dynamics between these constructs in both this population as well as others, and to assess the impact these dynamics may have in influencing care-seeking behaviors beyond serving as mediators.

## Conclusions

While previous research posits that concussion-related norms, knowledge, and attitudes influence behaviors, the results from our study suggest that that the relationship dynamics between these constructs are complex. Our findings indicate that concussion-related norms positively influence concussion-related knowledge and attitudes among parents of MS athletes. As such, there may be ways to develop and implement concussion education and prevention measures that target concussion-related norms, and in turn improve concussion related knowledge and attitudes. Taking this multifactorial approach may help to improve recognition and care of concussive injury in MS parents as well as others, ultimately leading to safer sport participation. The dataset for this study can be found in the S1 Dataset file.

## Supporting information

S1 Dataset (XLS)
